# Enhanced self-renewal of hematopoietic stem/progenitor cells mediated by the stem cell gene Sall4

**DOI:** 10.1186/1756-8722-4-38

**Published:** 2011-09-23

**Authors:** Jianchang Yang, Jerell R Aguila, Zaida Alipio, Raymond Lai, Louis M Fink, Yupo Ma

**Affiliations:** 1 Department of Cancer Biology, Nevada Cancer Institute, 1 Breakthrough Way, Las Vegas, NV 89135, USA; 2 Department of Pathology, SUNY at Stony Brook, Stony Brook, NY 11794, USA; 3 Department of Laboratory Medicine and Pathology, University of Alberta, Edmonton, Alberta T6G 2B7, Canada; 4 Biopharmaceutical Research Center of Chinese Academy of Medical Sciences & Peking Union Medical College, Suzhou, China

**Keywords:** Mouse Hematopoietic Stem Cell, Transplantation, Differentiation

## Abstract

**Background:**

Sall4 is a key factor for the maintenance of pluripotency and self-renewal of embryonic stem cells (ESCs). Our previous studies have shown that Sall4 is a robust stimulator for human hematopoietic stem and progenitor cell (HSC/HPC) expansion. The purpose of the current study is to further evaluate how Sall4 may affect HSC/HPC activities in a murine system.

**Methods:**

Lentiviral vectors expressing Sall4A or Sall4B isoform were used to transduce mouse bone marrow Lin-/Sca1+/c-Kit+ (LSK) cells and HSC/HPC self-renewal and differentiation were evaluated.

**Results:**

Forced expression of Sall4 isoforms led to sustained *ex vivo *proliferation of LSK cells. In addition, Sall4 expanded HSC/HPCs exhibited increased *in vivo *repopulating abilities after bone marrow transplantation. These activities were associated with dramatic upregulation of multiple HSC/HPC regulatory genes including HoxB4, Notch1, Bmi1, Runx1, Meis1 and Nf-ya. Consistently, downregulation of endogenous Sall4 expression led to reduced LSK cell proliferation and accelerated cell differentiation. Moreover, in myeloid progenitor cells (32D), overexpression of Sall4 isoforms inhibited granulocytic differentiation and permitted expansion of undifferentiated cells with defined cytokines, consistent with the known functions of Sall4 in the ES cell system.

**Conclusion:**

Sall4 is a potent regulator for HSC/HPC self-renewal, likely by increasing self-renewal activity and inhibiting differentiation. Our work provides further support that Sall4 manipulation may be a new model for expanding clinically transplantable stem cells.

## Background

Hematopoietic stem cells (HSCs) are rare cells defined by their unique ability to self-renew and their ability to replenish all blood cell types in the body. Under normal conditions however, only a small number of HSCs enter cell division to generate daughter cells and supply mature lineages. Thus a key question is how these HSCs are regulated for their self-renewal and multipotency properties. Scientists have tried to expand clinically transplantable HSCs *ex vivo*, mainly by optimizing the use of bioactive proteins and various hematopoietic cytokines. The few genes that have been reported to effectively expand HSCs *ex vivo *include the transcription factor homeobox B4 (HoxB4), Notch family receptors, as well as Wnt signaling proteins [1-3]. However, the long term outcome for clinical therapy using HSCs treated with these factors still needs to be further elucidated.

Sall4 is a zinc-finger transcription factor and is essential for developmental events [4,5]. We and others have previously reported that Sall4 plays important roles in maintaining the properties of embryonic stem cells (ESCs) by interacting with transcription factors Oct4 and Nanog [6-9]. In stem cells, Sall4 functions as both an activator and a repressor of gene transcription depending on the cell context. It suppresses important differentiation genes and activates key pluripotency genes [9,10]. Sall4 also plays positive roles in the reprogramming of differentiated cells to ESC-like cells, and generation of induced pluripotent stem cells (iPS)[11-13]. We and others have determined that Sall4 exists as two isoforms (Sall4A and Sall4B), and they have unique and overlapping functions [14-16]. Interestingly, Sall4 is one of the few genes that are also involved in adult tissue stem cells [17,18], and its protein expression is always correlated with the presence of stem and progenitor cell populations in various organ systems including bone marrow (BM) [16]. Importantly, SALL4 has been identified as a robust expanding factor for human HSCs [19]. We have recently studied the roles of Sall4 isoforms in controlling murine HSC/HPC properties. Our data indicate that a certain level of expression of Sall4 isoforms is necessary for normal HSC/HPC activity. Moreover, both Sall4A and Sall4B isoforms act as potent regulators of HSC/HPC self-renewal.

## Results

### Sall4 isoforms enhance and support murine LSK cell proliferation

Given the selective expression pattern of SALL4 proteins in hematopoietic stem/progenitor cell compartments [16], we asked whether increased expression of SALL4 isoforms may affect HSC/HPC phenotypes. To study this, mouse HSC/HPC s (Lin-, Sca-1 +, c-Kit +; LSK cells) were isolated and transduced with lentiviruses carrying either GFP alone (control), or together with Sall4A or Sall4B isoform. All GFP positive cells were determined by either fluorescence microscope inspection or flow cytometric analysis (Additional file 1: Figure. S1a, and data not shown). We next performed western analysis to assess the expression of Sall4 isoforms in infected NIH3T3 fibroblast cells. It was found that these cells expressed the appropriate Sall4A or Sall4B protein at 160 and 90 kDa. As negative control, the empty GFP vector did not generate Sall4A or Sall4B bands (Additional file 1: Figure. S1b).

**Figure 1:**
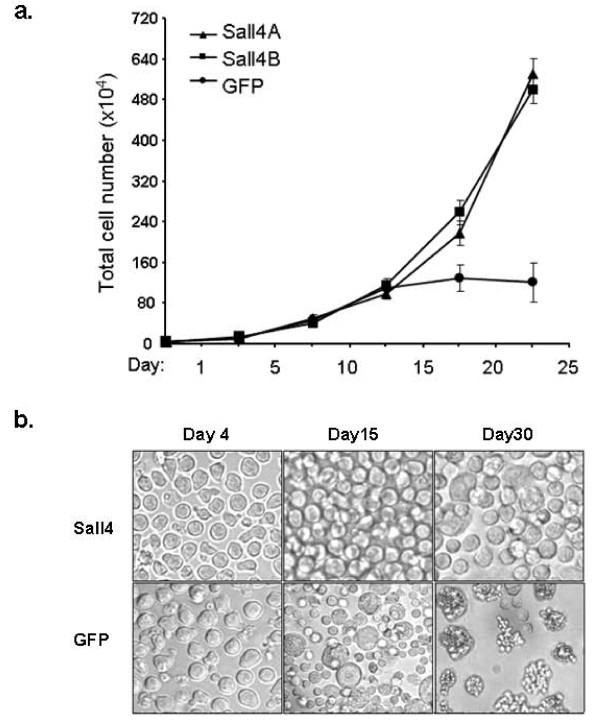
**Over-expression of Sall4 isoforms enhanced and supported LSK cell growth in culture**. (a) Viable cell numbers were counted for each group of LSK cells at different days (N = 4). Error bars represent standard error of the mean. (b) Morphology observation of GFP control or Sall4 isoforms transduced LSK cells under microscope inspection at different days. GFP transduced LSK cells grew relatively slower than Sall4 treated LSK cells and died after 4 weeks of culture, while the latter continued to proliferate in dishes.

**Figure 2:**
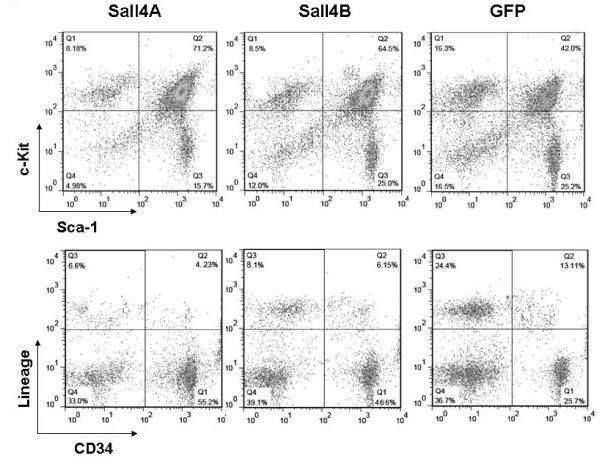
**Flow cytometry assays by using individual antibodies revealed that most Sall4A or Sall4B treated LSK cells retained immature surface phenotypes after 2 weeks culture, while GFP control LSK cells exhibited increased lineage differentiation**. The lineage cocktail consists of antibodies against CD5, CD45R, CD11b, Ter119, and GR-1. Experiments were repeated at least four times and a representative case is shown.

In culture, all LSK cells with and without Sall4 transduction were strictly cytokine dependent, which were not able to survive more than 5 days in the absence of cytokines (100 ng/ml mSCF, 6 ng/ml mIL-3 and 10 ng/ml hIL-6) or in each alone (data not shown). Thus, Sall4 overexpression did not convert cells to cytokine independence. In the combination of cytokines, the Sall4A or Sall4B transduced cells exhibited a ~2 fold increase in the rate of proliferation relative to the GFP control group at day 15 and thereafter (Figure 1a). During the second week, a small part of adherent cells were seen on the bottom of GFP control culture dishes, many floating cells resembled granulocytes and exhibited various shapes and different sizes, while these were barely observed in Sall4A or Sall4B infected cultures, in which cells were non-adherent, uniquely round, spherical and at similar size (Figure 1b). We performed flow cytometry assay at day 14 to test their surface antigens. It was found that most Sall4 treated HSC/HPCs retained immature surface phenotypes (Sall4A group: Sca-1+c-Kit+, 71.2% and Lin+, 10.8%; Sall4B group: Sca-1+c-Kit+, 64.5% and Lin+, 14.3%). By contrast, many GFP treated HSC/HPC cells exhibited differentiating markers (Sca-1+c-Kit +, 42% and Lin+, 37.5%) (Figure 2). After three weeks, the GFP treated LSK cells gradually stopped proliferating and were no longer viable after day 30. However, both Sall4A and Sall4B transduced cells continued to proliferate at a stable doubling time of about 48 hours and proliferated indefinitely in culture (> 4 months). These *in vitro *studies were repeated in two additional experiments and similar results were obtained.

To date, only several factors have been reported to expand HSC/HPCs *in vitro *while their stem cell characteristics were not impaired. To test whether Sall4- expanded HSC/HPCs may retain immature properties after long term culture, we repeated flow cytometry assays after 6 weeks of culture. We found that there were more than 70% of expanded cells retaining immature surface features (Sall4A group: Sca-1+, 79%, c-Kit +, 64% and Lin+, 21%; Sall4B group: Sca-1+, 75%, ckit+, 48% and Lin+, 22%). In addition, more than sixty percent of these cells were still Sca-1 positive even after 3 months culture (data not shown). These results indicate that overexpression of Sall4 isoforms is capable of enhancing and supporting primitive hematopoietic cells in the presence of combined cytokine stimulations.

### Enhanced HSC/HPC activity by forced expression of Sall4 isoforms

**Figure 3:**
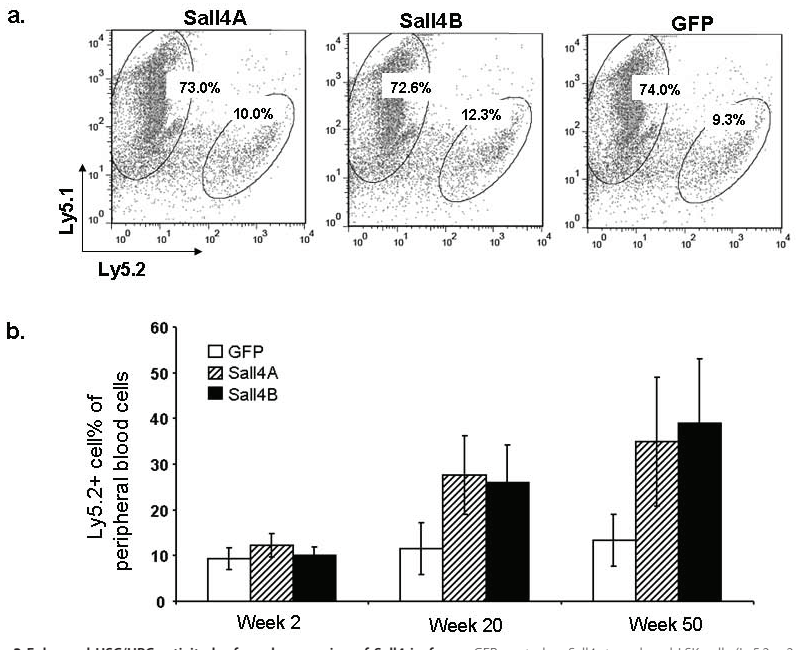
**Enhanced HSC/HPC activity by forced expression of Sall4 isoforms**. GFP control or Sall4 -transduced LSK cells (Ly5.2+, 2 × 10^6 ^cells, day-10) were transplanted into sublethally irradiated mice in combination with 1 × 10^5 ^normal BM cells with Ly5.1 phenotype. Four mice received GFP or Sall4A treated LSK cells and three mice received Sall4B treated LSK cells. (a) The population of donor cells (Ly5.2+) in the peripheral blood of the recipient mice was examined by flow cytometry assays with the indicated antibodies at 2 weeks after BM transfusion. (b) The Ly5.2+ cell percentages within the peripheral white blood cells of recipients were measured at 2, 20, and 50 weeks after transplantation of indicated lentivirus-infected BM cells. Error bars represent standard error of the mean.

**Figure 4:**
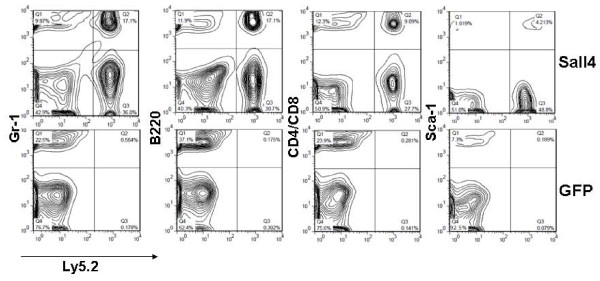
**The lineage (Gr-1, B220 and CD4/CD8) and primitive cell (Sca-1) distribution of Ly5.2+ cells was examined with the indicated antibodies for each group**. The data shown derive from three experiments with similar results.

Next, we conducted reconstitution assays to evaluate Sall4-expanded HSC/HPC function *in vivo*. After 10-day cultures, Sall4 or GFP treated cells were collected, and 2 × 10^6 ^cells (Ly5.2+) were intravenously transfused into sublethally irradiated (500 rad) Ly5.1 mice (GFP group, N = 4; Sall4A group, N = 4; and Sall4B group, N = 3). Two weeks after transfusion, the percentages of transplanted Ly5.2+ cells in peripheral blood mononuclear cells (PBMCs) were similar among all groups, as detected by flow cytometry assays with multiple lineage-specific antibodies (GFP group, 9.28%; Sall4A group, 10.0%; and Sall4B group, 12.3%, Figure 3a). However, the Ly5.2+ blood cells in recipient mice deriving from Sall4 transduced cells rose over months and surpassed the contribution from the GFP control-transduced HSC/HPCs by week 20 and week 50 (Figure 3b). Further more, these Ly5.2+ cells were able to reconstitute granulocyte, T cells, and B cells, as well as immature blood cells, as indicated by expression of GR-1, CD4/8, B220 and Sca-1 in BM cells respectively (Figure 4).

**Figure 5:**
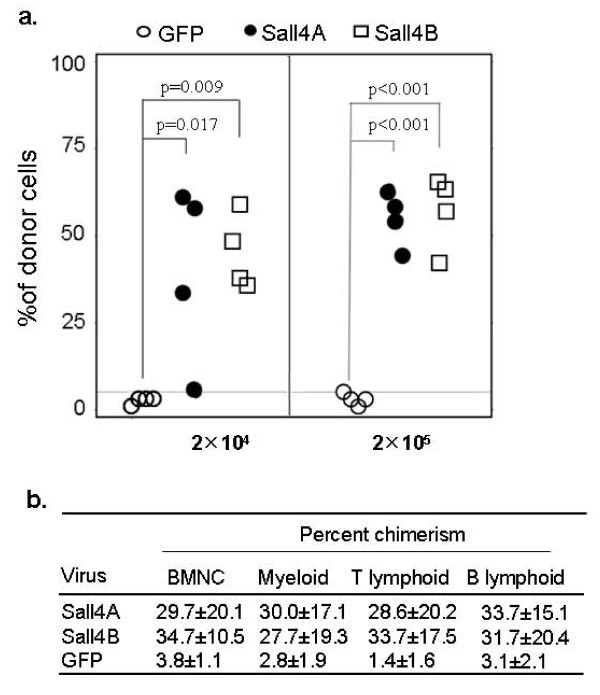
**Competitive repopulation assays were performed by using different amount of cultured cells (day-14) per recipient mouse**. (a) Three months after transplantation, the percentage of regenerated lineages contributed from donor cells which bore Ly5.2 antigen is plotted. (b) For each group, percent chimerism in each lineage is presented.

We further performed competitive repopulation assays to determine the activity of Sall4 transduced HSC/HPCs with day14 ex vivo cultured cells. Varying doses of cells baring Ly5.2 antigens were mixed with a constant number of non-transduced competitive BM cells (2 × 10^5^, Ly5.1+), and injected into lethally irradiated (900 rad) congenic mice (Ly5.1). After 3 months, the contributions of control or Sall4 transduced HSC/HPCs to the cellularities of mature lineages were measured by Ly5.2 antigen expression of peripheral blood cells. In each group, proportion of recipients exhibiting at least 5% donor-derived (Ly5.1+) leukocytes was determined and scored as positive only in which donor-derived (test) cells were detectable among B220+, CD4/CD8+ and Gr-1+ compartments. As shown in Figure 5a, when 2 × 10^4 ^donor cells were mixed with 2 × 10^5 ^competitive cells, Sall4A transduced LSK cells contributed to the repopulation of 3 of 4 recipients. Similarly, Sall4B transduced LSK cells contributed substantially to the repopulation of 4 of 4 recipients (Ly5.2+ cell percentage > 5%), whereas repopulation mediated by GFP control cells was not detected. When the number of infused cells was raised to 2 × 10^5 ^cells, both Sall4A and Sall4B transduced cells repopulated 4 of 4 recipients, whereas GFP transduced cells did not. Taken together, these data indicated that overexpression of Sall4 increased the repopulation activity of HSC/HPCs.

Of note, however, though HSC/HPCs were expanded by sustained ectopic expression of Sall4 isoforms and exhibited enhanced repopulating activity, when transplanted into lethally or sublethally irradiated syngeneic recipients, the mice grew normally and were apparently healthy. Analysis of percent chimerism of donor cells in each hematopoietic lineage confirmed that Sall4-transduced HSC/HPCs retained full differentiation capacity and no dramatic changes of lineage percentages were found (Figure 5b, 4 and data not shown).

To evaluate long-term safety issues such as myeloproliferative neoplasm (MPN)-like features, we transduced SALL4 isoforms to mouse LSK cells and then performed syngeneic transplantations to allow for long-term follow-up. As shown in Table 1, no MPN-like phenotypes were exhibited for more than 11 months (more than 50% lifespan in mice) or even more than 17 months post syngeneic transplantation (n = 18). Our studies are consistent with the finding that ex vivo expansion of HSC/HPCs mediated by SALL4 is still cytokine dependent, which is also consistent with that seen in HoxB4 mediated HSC/HPC expansion.

**Table 1:** Status of mice after being transplanted with Sall4 expanded cells

Strain	Number of mouse	Number of injected cells	Month post transfusion	Phenotype
C57B/6	4	Sall4A expanded cells, 2 × 10^6 ^	17	WT-like

B6.SJL	2	Sall4A expanded cells, 4 × 10^6^	15	WT-like

B6.SJL	4	Sall4A expanded cells, 6 × 10^6^	11	WT-like

B6.SJL	3	Sall4B expanded cells, 4 × 10^6^	15	WT-like

B6.SJL	5	Sall4B expanded cells, 6 × 10^6^	11	WT-like

B6.SJL	2	GFP-infected cells, 4 × 10^6^	15	WT-like

B6.SJL	7	GFP-infected cells, 6 × 10^6^	11	WT-like

### Down-regulation of Sall4 accelerated differentiation of HSC/HPCs

**Figure 6:**
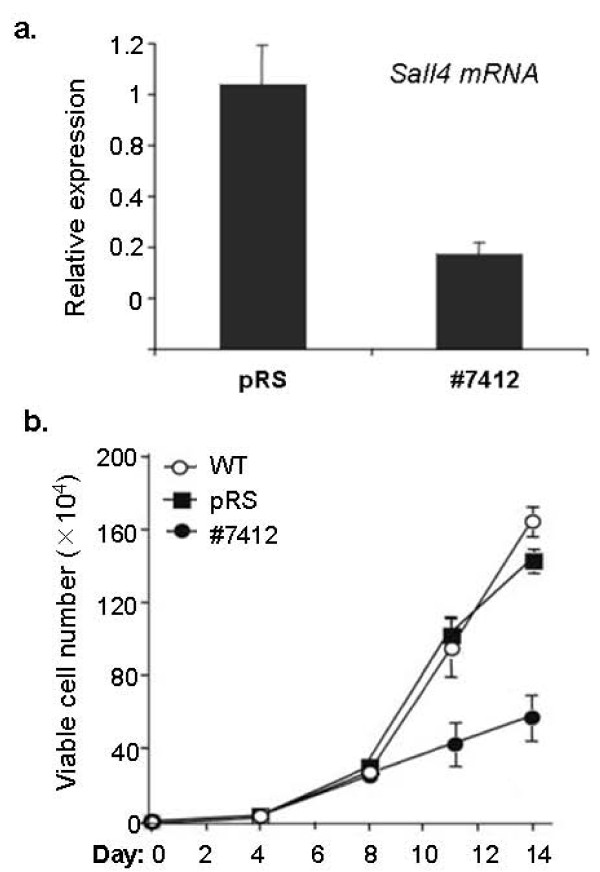
**Sall4 reduction induced decreased LSK cell proliferation and accelerated differentiation**. Freshly isolated BM LSK cells were cultured and infected with pRS control retrovirus or expressing *Sall4*-specific short-hairpin RNA (#7412). Cells were grown with puromycin (1.2 μg/ml) selection for 10 days. (a) Total RNA was isolated from cells and subjected to qRT-PCR analysis to determine relative Sall4 mRNA expression levels in control and #7412 treated cells. (b) Viable cell numbers were counted for each group at different days. The data shown represent three independent replicates. Error bars represent standard error of the mean.

**Figure 7:**
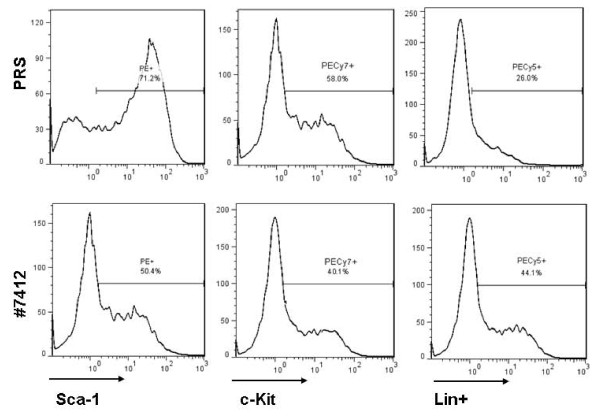
**Flow cytometry assay was performed using anti-Sca1, anti-cKit or lineage antibody cocktail for each group at day10**. A representative case is shown.

We previously showed that Sall4 was exclusively expressed in CD34 positive hematopoietic stem/progenitor cells [16]. To further test the effect of Sall4 in LSK cells, we performed a Sall4 knockdown study in isolated mouse BM LSK cells by an RNA interference strategy as previously described [20]. After 10 days culture under puromycine selection (1.2 μg/ml), a ~62% reduction of Sall4 mRNA levels by #7412 expressing retrovirus was confirmed by qRT-PCR (Figure 6a). We found that Sall4-reduction LSK cells exhibited a decreased proliferation rate relative to the pRS control or non-treated cells (Figure 6b). In addition, reduction of endogenous Sall4 in LSK cells caused increased lineage differentiation markers (Sca-1+: 48 ± 7% versus pRS control: 63.3 ± 9%; cKit+:40 ± 6% versus pRS control: 54 ± 6%, and Lin+:42 ± 3% versus pRS control: 25 ± 3%, N = 3) during the two weeks of culture in dishes (Figure 7). These results suggest that proper expression of endogenous Sall4 is required for the self-renewal activity of the HSC/HPCs.

### Sall4 up-regulates multiple important HSC/HPC regulators

**Figure 8:**
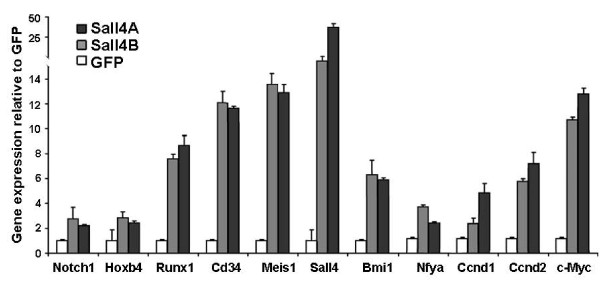
**Sall4A or Sall4B overexpression induces the expression of important HSC regulatory genes in cultured LSK cells**. Murine Lin-Sca-1+ cells were infected with lentiviruses each containing Sall4 isoform or GFP control, cultured for 7 days, and GFP+ LSK cells were isolated by FACS. Total RNA was extracted and mRNA for the indicated genes was measured by qRT-PCR. Error bars represent standard error of the mean (N = 3).

We have mapped SALL4 global gene targets using chromatin-immunoprecipitation followed by microarray hybridization (ChIP-chip) in myeloid leukemic NB4 cells as well as normal human CD34+ BM cells ([20], and unpublished data). Bmi1, TPO, Runx1, c-Myc, CD34, Meis1 and Nf-ya were identified as potential Sall4 downstream targets in both cell types. To further test if Sall4 is able to regulate these gene expressions, we performed qRT-PCR assays. We detected mRNA levels of Sall4 isoforms as well as some important HSC/HPC regulatory genes at day 7 post lentiviral transduction. Cells were collected and total RNAs were prepared and subjected to qRT-PCR assays. Results showed that in Sall4A or Sall4B transduced LSK cells, there were ~40 and ~20 fold increases of relevant Sall4A or Sall4B mRNA levels as compared with GFP control transduced cells, (this further confirms a successful Sall4 lentiviral transduction). Of note, transcripts of Bmi1, HoxB4, Notch1, Runx1, CD34, Meis1 and Nf-ya were all significantly activated, exhibiting a 2.5~15 fold up-regulation when cells were treated with Sall4 isoforms (Figure 8). ALL of these genes have been reported to play active roles in maintaining short term or long term HSC proliferation. In addition, Sall4 isoforms dramatically stimulate several molecules that are implicated in the Wnt/beta-catenin signaling pathway which is essential for HSC growth, including c-Myc, cyclin D1 and cyclin D2.

### Sall4 inhibits myeloid progenitor differentiation

**Figure 9:**
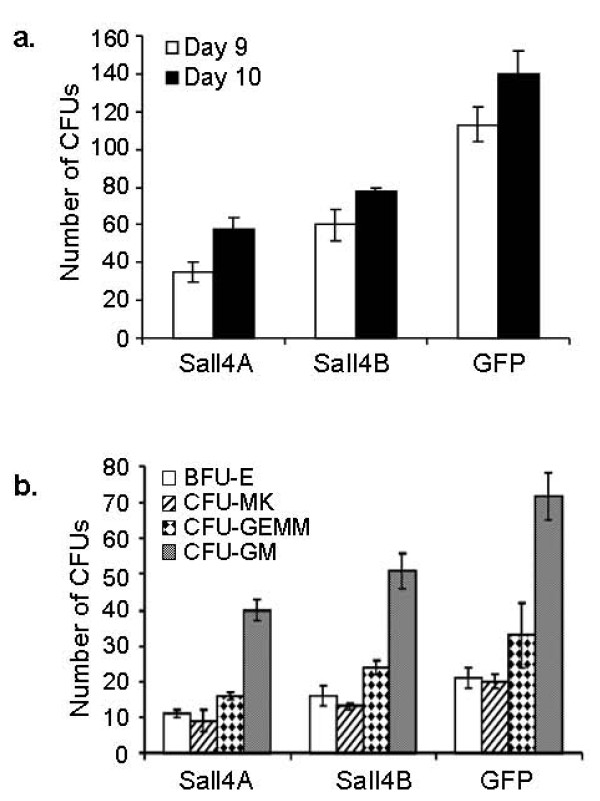
**CFU assays of Sall4-transduced BM cells**. BM cells were isolated and infected with GFP control or Sall4 expressing lentivirus. Four days later, 10^4 ^cells per dish were plated and colony numbers (> 100 cells) were counted at different days (a). Various CFU types were recorded at day 12 (b).

We have performed *in vitro *colony forming assay to evaluate effects of Sall4 isoforms on hematopoietic cell growth and progenitor differentiation. Mouse BM cells were isolated and infected with Sall4- expressing lentivirus. After four days, 10^4 ^cells were mixed with MethoCult^® ^medium and seeded in dishes for colony formation. As shown in Figure 9a and 9b, both Sall4A and Sall4B transduced cells could generate various colonies including CFU-GM, CFU-GEMM, and BFU-E since day 9. However, the total numbers of each type of colony are lower than that from the GFP control infected cells. Two additional experiments with various initial seeding cell numbers showed similar results (data not shown). These experiments indicate that over-expression of Sall4 isoforms down-modulate myeloid differentiation versus proliferation from early primitive cells.

**Figure 10:**
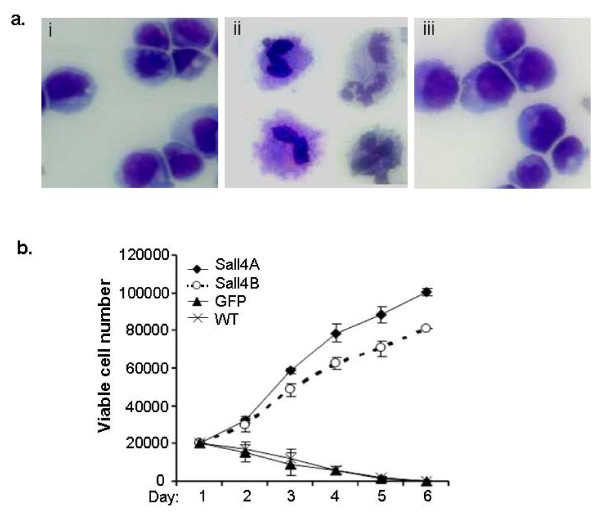
**Overexpression of Sall4 immortalized 32D cells and blocked G-CSF dependent differentiation**. (a)Wright-Giemsa staining of 32D cells shows morphology of 32D cells with IL-3 alone (i), with G-CSF alone (ii), or transduced with Sall4A and with G-CSF alone (iii). (b) Growth curves of Sall4-32D cells cultured with G-CSF alone. In cells that were transduced with Sall4A or Sall4B, a 5 or 4-fold increase in the number of viable cells was observed from day 1 to 6.

We next extended our study to a myeloid progenitor cell line (32D), to determine how Sall4 may affect hematopoietic progenitor cell differentiation. 32D cells were cultured and infected with empty GFP control or Sall4 expressing lentiviruses. The transduction efficiency was more than 80% as judged by fluorescent observation as well as flow cytometry assays (Additional file 2: Figure S2). During culture, we found that the 32D cells with or without transduction of Sall4 isoforms are all strictly IL-3 dependent, which are unable to grow when removed from IL-3. Thus, over-expression of Sall4 isoforms did not convert the 32D cells to cytokine independence. In agreement with previous reports [21,22], 32D cells proliferate as undifferentiated blasts when maintained in IL-3, but differentiate into mature granulocytes when stimulated with G-CSF (Figure 10a. i, ii). However, when cultured with G-CSF but in the absence of IL-3, control 32D cells died after 5-6 days. By contrast, the Sall4 A or Sall4 B transduced 32D cells proliferated at a 3 to 6-fold higher rate than the control counterparts in the same media and then grew indefinitely (Figure 10b). In addition, these cells retained undifferentiated blast morphology in the presence of G-CSF but lack of IL-3 (Figure 10a, iii). This study indicates that constitutive expression of Sall4 inhibits G-CSF induced granulocytic differentiation and permits expansion of undifferentiated cells in 32D myeloid progenitors.

## Discussion

We recently reported that SALL4 is a robust expanding factor for human HSC/HPCs [19]. In the current study, we demonstrate that both Sall4A and Sall4B isoforms also stimulate sustained cell proliferation of murine HSC/HPCs (> 4 months in culture) while their capacity of multi-lineage differentiation was not impaired. Similarly, when Sall4 expanded cells were transfused into irradiated mouse recipients, no abnormal hematopoietic or leukemic features were observed even after 17 months of BM transplantation (n = 18). This study is consistent with the finding that HSC/HPC expansion mediated by Sall4 is still under cytokine control. In fact, in our previous transgenic mouse studies of Sall4B overexpression controlled by a universal CMV promoter, there was an increased incidence of leukemic formation in older mice [16]. Similar studies with Sall4A overexpression under the same promoter, however, were free of leukemic formation (N = 170, five transgenic mouse lines, data not shown) after 2 years of observation. This could be due to an abnormal niche resulting from dysregulated SALL4B expression in various mouse tissues throughout the mouse development with this universal CMV promoter and/or SALL4B may bear oncogenic potential.

In isolated mouse LSK cells, forced expression of Sall4 isoforms dramatically stimulate multiple known HSC regulators, such as Notch1, HOXB4, cMyc, CyclinD1, CyclinD2, Bmi1, Nfya, Runx1and Meis1. This is of great interest since all of these factors play important roles in regulating HSC activity [23-26]. Specifically, Notch1 and HOXB4 are thought to be highly interesting candidates for therapeutic stem cell expansion [27]. cMyc may act as a downstream mediator of both factors in murine HSCs, while the trimetric transcription factor Nfya is able to activate multiple HSC regulatory genes including HOXB4 [28,29]. The transcription factors Meis1, Runx1 and the poly comb gene Bmi1 are all expressed at high levels in HSCs and required for cell activity [30-32]. Though whether/how Sall4 directly regulates other factors is unresolved, in our previous ChIP-chip study on mouse ESCs, Sall4 bound approximately twice as many annotated genes within promoter regions as Nanog and approximately four times as many as Oct4 [7]. Moreover, Sall4 seems to act as a "central tower of pluripotency" in ES cells in association with Oct3 ⁄ 4, Sox2 and Nanog [33,34]. Further detailed studies are required to elucidate whether it may also function as a master controller in modulating genetic networks within the HSC system.

Another discovery is the finding of how overexpression of Sall4 isoforms affects G-CSF induced granulocytic differentiation. The results obtained here were similar as compared with the Sall4 activities in the human system [19]. We have hypothesized that the Sall4 gene is also involved in mediating cell fate decisions during hematopoiesis, helping to regulate the exquisite balance among self-renewal, differentiation, and proliferation required for normal blood formation. Our findings demonstrate that sustained activation of both Sall4A and Sall4B isoforms inhibits granulocytic differentiation of 32D myeloid progenitors, supporting the view that Sall4 is capable of influencing cell fate determination in hematopoietic cells. This may also explain, at least in part, why Sall4 isoforms are expressed preferentially in HSCs, down-regulated rapidly in HPCs, and absent in the differentiated lineage populations.

In summary, our work demonstrates that the potent stem cell factor Sall4, which is preferentially expressed within the HSC/HPC pool during hematopoiesis, activates and integrates multiple genetic pathways responsible for the precisely controlled proliferation and differentiation of HSC/HPCs. Sall4 is thus an excellent candidate for the genetic manipulation of HSC/HPC self-renewal *in vivo *and *in vitro*.

## Methods

### Mice

C57Bl/6J (Ly5.2) and congenic C57Bl/6.SJL-Ly5.1-Pep3b (Ly5.1) mice at 8-12 weeks of age were obtained from The Jackson Laboratory (Bar Harbor, ME). All animal experiments were preapproved by the Office of Laboratory Animal Welfare, Institutional Animal Care and Use Committee.

### Gene cloning and lentiviral transduction

The full-length cDNA of mouse Sall4A and Sall4B were cloned into the SalI/NotI site of entry vector pENTR3C (Invitrogen, Carlsbad, CA). A Gateway LR reaction was carried out to subclone the cDNA into the lentiviral mammalian expression vector pDEST-CMVFG12 [35], which contains reading frame of the enhanced green fluorescent protein (EGFP). The obtained lentiviral constructs were confirmed by PCR reaction, enzyme digestion and gene sequencing. To generate lentivirus, the expression vectors were transfected into 293FT packaging cells (Invitrogen) along with pSPAX2 and pMD2.G plasmids (Addgene Inc., Cambridge, MA) for 48 hrs, then pooled filtered supernatants were used to infect cells in the presence of polybrene (8 μg/ml, Millipore, Billerica, MA).

For lentivirus transduction, the isolated LSK or Lin-/Sca-1+ cells were cultured in Dulbecco's modified Eagle's medium supplemented with 10% fetal bovine serum (FBS) in the presence of mSCF (100 ng/ml), mIL-3 (6 ng/ml) and hIL-6 (10 ng/ml). The cells (~1 × 10^6 ^per ml) were then incubated with high titer lentivirus (~1 × 10^6 ^per ml) for 24 hours before being replaced with fresh media. For cultured 32D cells, lentivirus was added in conditioned medium (RMI1640 supplemented with 10% FBS) in the presence of mIL-3 (1 ng/ml) for 24 hours, then fresh media was used thereafter. Cytokines were purchased from ProSpec-Tany TechnoGene Ltd (Rehovot, Israel).

### SDS-PAGE and western blotting

Total cell lysates prepared from 1 × 10^5 ^wide type or lentiviral infected cells were electrophoresed through 7% SDS-polyacrylamide gels, transferred to nitrocellulose membranes, and then immunoblotted using the anti-Sall4 monoclonal antibody (Abcam, Cambridge, United Kingdom). Immunostained proteins were detected using enhanced chemilluminescence blot reagents (Thermo Fisher Scientific, Rockford, IL). Blots were detected by a Kodak Image Station 2000 MM (Kodak, Rochester, NY).

### Isolation of mouse BM hematopoietic stem/progenitor cells

Cells were isolated from whole BM by immunostaining with either magnetic bead isolation or fluorescence-activated cell sorting. To obtain BM, 8*- *to 12*-*week-old mice were euthanized by carbon dioxide inhalation and the femurs and tibias removed. A 25-gauge needle was used to expel the marrow by a buffer solution contained phosphate-buffered saline (PBS), pH 7.2, 0.5% bovine serum albumin (BSA), and 2 mM EDTA. For depletion of mature hematopoietic cells, the Lineage Cell Depletion Kit (Miltenyi Biotec, Bergisch Gladbach, Germany) was used. The lineage (CD5, CD45R, CD11b, Ter119, and GR-1) negative cells were collected through mini MACS separation columns (Miltenyi Biotech) while in a magnetic field. For positive purification, the collected lineage negative cell fraction were dual stained with fluorescein isothiocyanate (FITC) conjugated anti Sca-1 and anti-FITC MicroBeads (Miltenyi Biotech) and separated again to yield HSC/HPCs (Lin-/Sca-1+). At this step, we confirmed that more than 95% of the separated cells were Lin-/Sca-1+ cells by flow cytometric analysis.

In some cases, fluorescence-activated cell sorting of Lin-/Sca-1+/c-Kit+ (LSK) was performed on the Reflection Cell sorter (iCyt, Champaign, IL). Antibodies were purchased from BD Pharmingen (San Diego, CA). Cells sorted were propidium iodide (PI)-negative, Lin (CD5, B220, Ter119, Mac1, and Gr-1) negative, c-Kit, Sca-1 and GFP positive.

### Preparation of peripheral blood

Peripheral blood was obtained from each mouse by tail vein bleeding. One hundred microliters of blood was incubated for 10 minutes at room temperature with 3 mL ice-cold erythrocyte lysing solution (150 mM NH4Cl, 10 mM NaHCO3, 1 mM EDTA, pH 7.4), washed with PBS and resuspended in PBS and 1% paraformaldehyde (Sigma, St Louis, MO) and kept at 4°C until analysis.

### Immunostaining and flow cytometry

Freshly isolated cells or cultured cells were stained by described fluorescence-labeled antibodies. The flow cytometry data were collected by using a FACScan or FACSCalibur machine (Becton Dickinson, Franklin Lakes, NJ) and analyzed by using FLOWJO or CELLQUEST software.

### RNA interference (RNAi) and quantitative reverse transcription (qRT-PCR) Analyses

RNAi mediated Sall4 knockdown and qRT-PCR analyses were performed as reported previously [20]. The primer sets are described in Additional file 3: Table S1.

### 32D cell culture and induction of differentiation

The 32D cells were cultured as previously described [36]. For granulocytic differentiation, IL-3 was removed by washing cells 3 times, then G-CSF (R&D Systems, Minneapolis, MN) was added to cells at a final concentration of 200 ng/ml.

### CFU assay of BM cells

Tubes of MethoCult^® ^GF M3434 (Stem Cell Technologies, Vancouver, BC, Canada) medium were thawed overnight in a 4°C refrigerator. Sall4 or GFP-transduced BM cells were prepared at 10 × the final concentration required. Cell suspensions of 1 × 10^5 ^cells per mL were prepared and 0.3 mL of cells were added to 3 mL of MethoCult^® ^medium for duplicate cultures. 1.1 mL of cells was dispensed per 35 mm dish. The cells were incubated for 8-12 days at 37°C with 5% CO_2 _and ≥95% humidity. The BFU-E, CFU-GM and CFU-GEMM colonies were observed with bright field and fluorescent microscopy. CFUs were counted under the microscope 9 days after the cells were plated in MethoCult^® ^medium. A colony with more than 100 cells was counted as a positive colony.

## Competing interests

The authors declare that they have no competing interests.

## Authors' contributions

All authors are accountable for the integrity of the research results. JY and YM are responsible for the conception of the research and JY, ZA, JRA are responsible for the execution and for data collection; JY, LMF, RL and YM wrote the paper with contributions from the other authors. All authors read and approved the final manuscript.

## Support and financial disclosure declaration

This work is supported in part by Leukemia & Lymphoma Society Special Fellow Award (3366-09) (J.Y.), Department of Defense Grant W81XWH-10-0046 (LMF), and National Institutes of Health Grant NIH R01HL087948 (Y.M.).

Financial Disclosure Declaration: Y.M. is a scientific consultant to MarrowSource Therapeutics International LLC.

## Supplementary Material

Additional file 1 **Figure. S1. Transduction of GFP or Sall4 isoform- expressing lentiviruses in mouse BM LSK cells or NIH3T3 fibroblasts**. (a) Mouse BM Lin-Sca-1+c-Kit (LSK) cells were isolated and infected with lentivirus as described in Methods. Images were taken 72 hours post infection, bright field (up) and fluorescent (bottom) images illustrating the infection efficiency of lentiviral constructs containing GFP + Sall4A, GFP + Sall4B or GFP only. (b) A western blot analysis was performed to confirm the expression of Sall4 isoforms in lentivirus transduced NIH3T3 cell lines using anti-Sall4 antibodies. Click here for file

Additional file 2 **Figure S2. Transduction of GFP control or Sall4 isoform- expressing lentiviruses in 32D cells**. (a) Bright field (up) and fluorescent (bottom) images illustrating the infection efficiency of lentiviral constructs containing GFP + Sall4A, GFP + Sall4B or GFP only. Images were taken 72 hours post infection with lentiviruses. (b) Flow cytometric assessment of virus infected cells by using anti-FITC antibody 5 days post infection. Click here for file

Additional file 3 **Table S1. Primer information used for qRT-PCR**. Click here for file
