# Supplementary figures and images for: Enhanced self-renewal of hematopoietic stem/progenitor cells mediated by the stem cell gene Sall4
Source: J Hematol Oncol. 2011 Sep 23;4:38. doi: 10.1186/1756-8722-4-38 (PMC3184628; doi:10.1186/1756-8722-4-38)

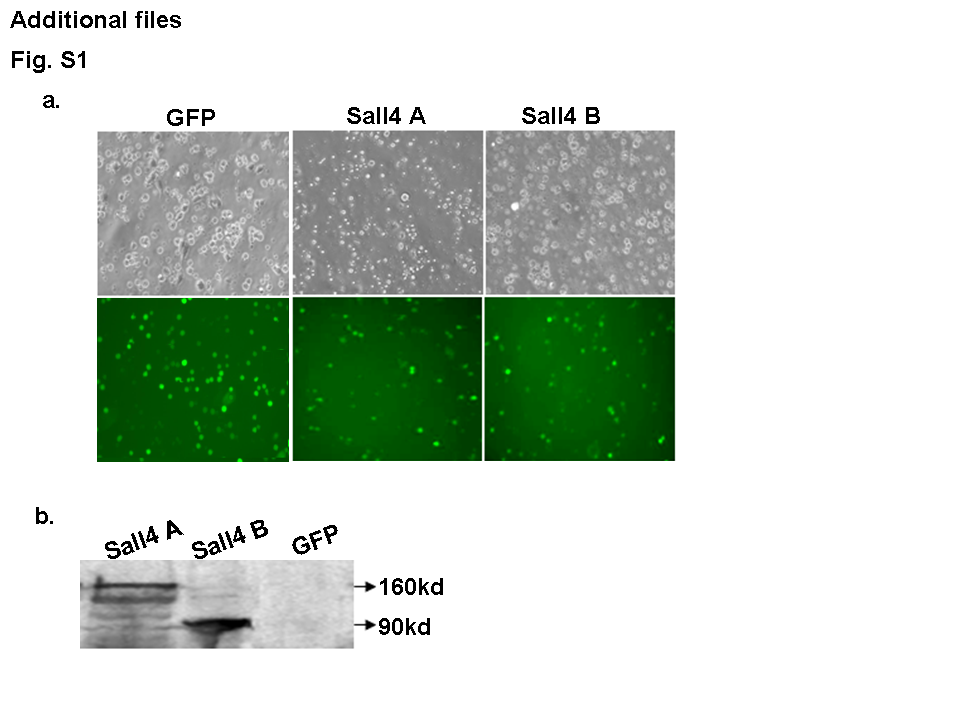

Supplement: Additional file 1 — Figure. S1. Transduction of GFP or Sall4 isoform- expressing lentiviruses in mouse BM LSK cells or NIH3T3 fibroblasts. (a) Mouse BM Lin-Sca-1+c-Kit (LSK) cells were isolated and infected with lentivirus as described in Methods. Images were taken 72 hours post infection, bright field (up) and fluorescent (bottom) images illustrating the infection efficiency of lentiviral constructs containing GFP + Sall4A, GFP + Sall4B or GFP only. (b) A western blot analysis was performed to confirm the expression of Sall4 isoforms in lentivirus transduced NIH3T3 cell lines using anti-Sall4 antibodies. [file 1756-8722-4-38-S1.TIFF]

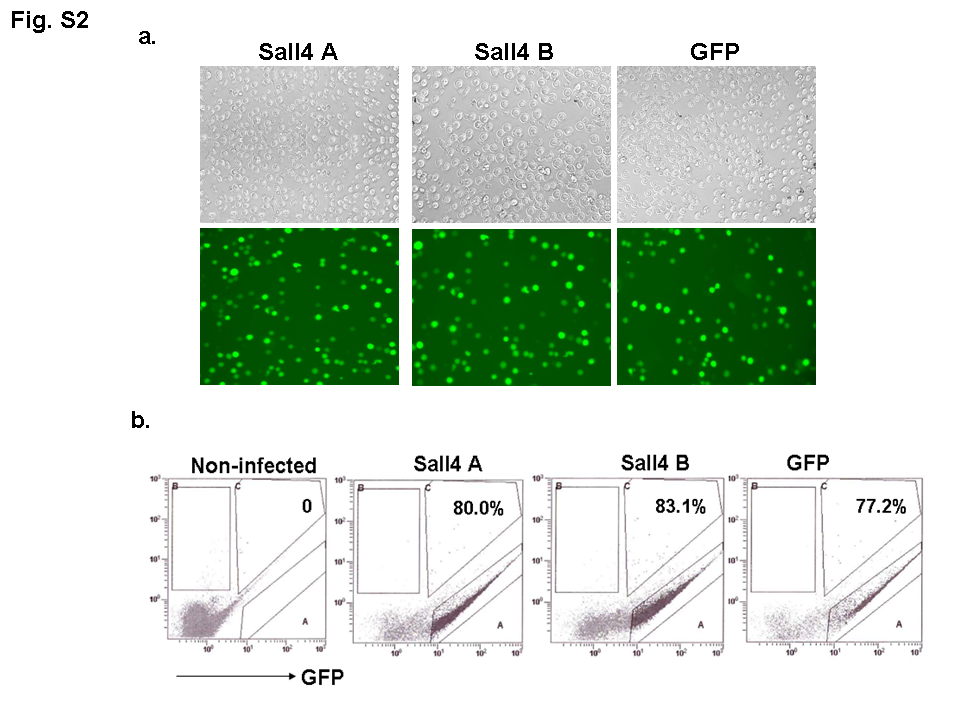

Supplement: Additional file 2 — Figure S2. Transduction of GFP control or Sall4 isoform- expressing lentiviruses in 32D cells. (a) Bright field (up) and fluorescent (bottom) images illustrating the infection efficiency of lentiviral constructs containing GFP + Sall4A, GFP + Sall4B or GFP only. Images were taken 72 hours post infection with lentiviruses. (b) Flow cytometric assessment of virus infected cells by using anti-FITC antibody 5 days post infection. [file 1756-8722-4-38-S2.TIFF]
