# Supplementary material for: Enhanced self-renewal of hematopoietic stem/progenitor cells mediated by the stem cell gene Sall4
Source: J Hematol Oncol. 2011 Sep 23;4:38. doi: 10.1186/1756-8722-4-38 (PMC3184628; doi:10.1186/1756-8722-4-38)
Supplement: Additional file 3 — Table S1. Primer information used for qRT-PCR. [file 1756-8722-4-38-S3.DOC]

**Additional file 3**

**Table S1. Primers used for qRT-PCR**

| **Sequence** | **Primer** |
| --- | --- |
| CCTCTCCTGCTACCGCACAACGCAC | Ccnd1 F |
| CTCTCAGGGTGATGCAGATTCTATCTC | Ccnd1 R |
| CTGTGCATTTACACCGACAAC | Ccnd2 F |
| CACTACCAGTTCCCACTCCAG | Ccnd2 R |
| TTTGTCTATTTGGGGACAGTGTT | cMyc F |
| CAGCTTCTCCGAGACCAGCTTGGCAGC | cMyc R |
| TGGAACACAAGGTTTACAGCAG | Nfya F |
| CTGCCCACCCTGAATCTGG | Nfya R |
| CTCCGTGCTACCCACTCACT | Runx1 F |
| ATGACGGTGACCAGAGTGC | Runx1 R |
| AAACCAGACCACTCCTGAAC | Bmi1 F |
| TCTTCTTCTCTTCATCTCATTTTTG | Bmi1 R |
| CACTGCTTCCTGGTACCAGTGG | Notch1 F |
| AGGATGACTGCACACATTGC | Notch1 R |
| CTGGATGCGCAAAGTTCAC | Hoxb4 F |
| GTGAAACTCCTTCTCCAACTCC | Hoxb4 R |
| CGCTTTAAAGAGAGATAAAGATGC | Meis1 F |
| GTTCTGGATTAGAAGAGAATAGAGG | Meis1 R |
| GGCAGCGGTACACACCTTGTGG | Tpo F |
| CATCAGGACCCAGGATTTTG | Tpo R |
| GCCACCAGAGCTATTCCCGAAAG | Cd34 F |
| TTTTCTTCCCAACAGCCATC | Cd34 R |
| GCAGCCTCAGCAGCTACC | Sall4 F |
| GGGGAGTTCACTGGAGCAC | Sall4 R |
